# Simultaneous Measurement of Thermal Conductivity and Volumetric Heat Capacity of Thermal Interface Materials Using Thermoreflectance

**DOI:** 10.1021/acsaelm.4c00691

**Published:** 2024-06-27

**Authors:** Zeina Abdallah, James W. Pomeroy, Nicolas Blasakis, Athanasios Baltopoulos, Martin Kuball

**Affiliations:** † Center for Device Thermography and Reliability (CDTR), University of Bristol, Bristol BS8 1TL, United Kingdom; ‡ Adamant Composites Ltd., Agias lavras & Stadiou Str., Platani-Patras, Achaia GR-26504, Greece

**Keywords:** thermal interface material, frequency-domain thermoreflectance, thermal characterization, thermal conductivity, volumetric heat capacity, package assembly, thermal management

## Abstract

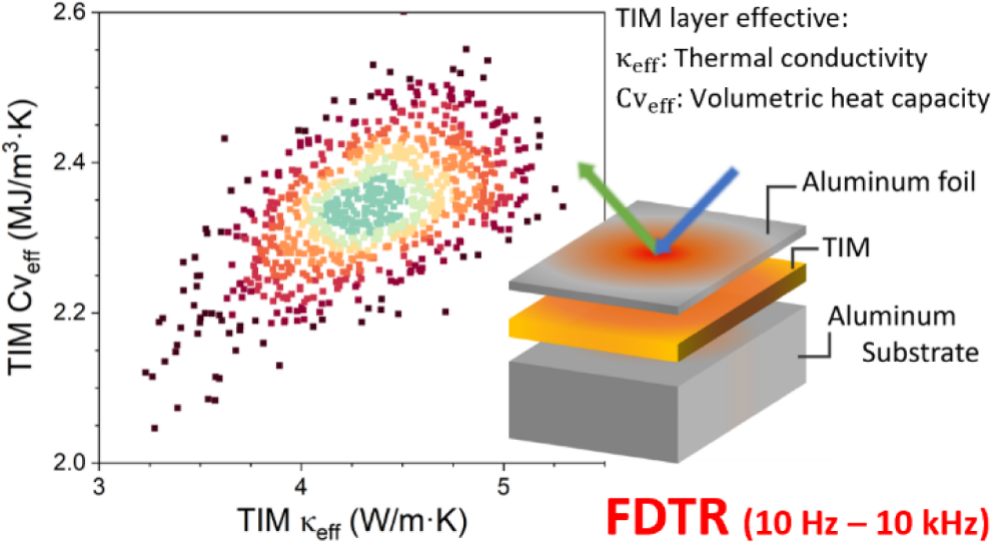

Thermal interface materials are crucial to minimize the thermal resistance between a semiconductor device and a heat sink, especially for high-power electronic devices, which are susceptible to self-heating-induced failures. The effectiveness of these interface materials depends on their low thermal contact resistance coupled with high thermal conductivity. Various characterization techniques are used to determine the thermal properties of the thermal interface materials. However, their bulk or free-standing thermal properties are typically assessed rather than their thermal performance when applied as a thin layer in real application. In this study, we introduce a low-frequency range frequency domain thermoreflectance method that can measure the effective thermal conductivity and volumetric heat capacity of thermal interface materials simultaneously in situ, illustrated on silver-filled thermal interface material samples, offering a distinct advantage over traditional techniques such as ASTM D5470. Monte Carlo fitting is used to quantify the thermal conductivities and heat capacities and their uncertainties, which are compared to a more efficient least-squares method.

## Introduction

Thermal management is increasingly important for high-power density electronic devices.^1^ Thermal dissipation within device packages is limited by the thermal conductivity of the packaging and thermal interface materials (TIMs).^2,3^ TIMs encompass thermal greases, solders, gap fillers, thermal adhesives, and gels^4−8^ and can contribute significantly to the total device thermal resistance. TIMs are therefore a key element to optimize for improved thermal management of packaged devices, evidenced by the rapid growth of the TIM market.^9^ High thermal conductivity (i.e., low thermal resistance), good processability, and good dielectric (or high electrical conductivity, depending on the application) properties are desirable characteristics of TIMs.^10,11^ Aside from the thermal conductivity of TIMs, the thermal contact resistance also needs to be considered and minimized because it contributes to the total thermal resistance.^12^ Selecting the right TIM for a specific application is challenging, not only due to the different options available but also because of the lack of unanimously commonly approved thermal test methodologies,^13^ especially which are representative of the thermal properties achievable in real applications. A commonly used steady-state test standard is ASTM D5470,^11,14−16^ which measures the thermal resistance and therefore the bulk thermal conductivity of TIMs. However, ASTM D5470 does not fully represent the actual use of the TIM in a real application, because factors such as nonuniform surface heating, nonuniform pressure loading, and dissimilar mating surfaces can affect the TIM thermal resistance.^6^ Therefore, there can be a significant inconsistency when attempting to reproduce manufacturer’s datasheet values.^6,7^

Low-frequency range frequency domain thermoreflectance (FDTR) has recently been demonstrated as an effective in situ thermal conductivity measurement technique for layered electronic device structures, suitable for layer thicknesses ranging from tens of micrometers to several millimeters.^17−20^ Adjusting the FDTR modulation frequency range (10 Hz to 10 kHz) allows the thermal penetration depth to be adjusted continuously, i.e., probing the thermal properties of layers at different depths within the structure being tested. Here, we demonstrate that low-frequency FDTR can be applied to TIM thermal conductivity measurements, while simultaneously determining its volumetric heat capacity. Volumetric heat capacity is another important property needed for both transient device thermal simulation and analyzing the results of transient thermal conductivity measurement techniques.^4,21^ Previously, the 3-omega method had been used to measure both the TIM thermal conductivity and the volumetric heat capacity.^22^ However, this method has uncertainties for volumetric heat capacity as high as ±30%, and it requires specific sample preparation. Differential scanning calorimetry (DSC) is the standard way to measure volumetric heat capacity, but it requires separate sample preparation from thermal conductivity measurements.^21^ In principle, FDTR could be used to obtain thermal conductivities and volumetric heat capacities in a single analysis since both properties affect transient heat flow through a structure. However, the ability of the low-frequency range FDTR to determine simultaneously the effective thermal conductivity () and the effective volumetric heat capacity () of a TIM film is yet to be demonstrated, which is the focus of this work; two silver filler-based TIM samples from different suppliers are measured to highlight this capability.

## Experimental Details

The principle of the low-frequency range FDTR system used in this study is described in ref. (17). The FDTR method is based on an optical pump–probe configuration, where the pump laser diode (450 nm) is modulated by a function generator to periodically heat the sample surface at a set frequency between 10 Hz and 10 kHz. The probe laser (520 nm) is used to monitor the surface temperature change Δ*T* of the transducer, which is proportional to the relative change in reflectivity Δ*R* of the transducer, .^17,23^ The phase response of the reflected signal is measured as a function of the modulation frequency, and a heat diffusion model is fitted to the experimental data to obtain unknown thermal properties of single or multiple layers.^17,24^ Both the pump and probe spot 1/*e*^2^ radius have been chosen at around 368 ± 16 μm, i.e., average thermal properties are measured over this spot size. The laser’s spot radius was determined using a 5 mm-thick pure silicon sample with precisely known thermal properties.^17^ A 150 nm gold transducer with a 10 nm chromium (Cr) adhesion layer was deposited onto the TIMs studied. The high thermoreflectance coefficient of gold (*C*_TR_ = 2.3 × 10^–4^ K^–1^) at the chosen 520 nm probe laser ensures a high measurement sensitivity.^25^ The accuracy of this technique was demonstrated on a range of reference materials, including a 0.25 mm-thick CVD diamond.^17^ Furthermore, the accuracy was verified by comparing the FDTR-measured thermal conductivity of a clad metal–diamond composite with the values obtained from the flash method.^19^ This comparison showed good consistency between these methods within their corresponding error bars.^19^ In this study, FDTR is used to measure both the  and  parameters of TIM films.

**Figure 1:**
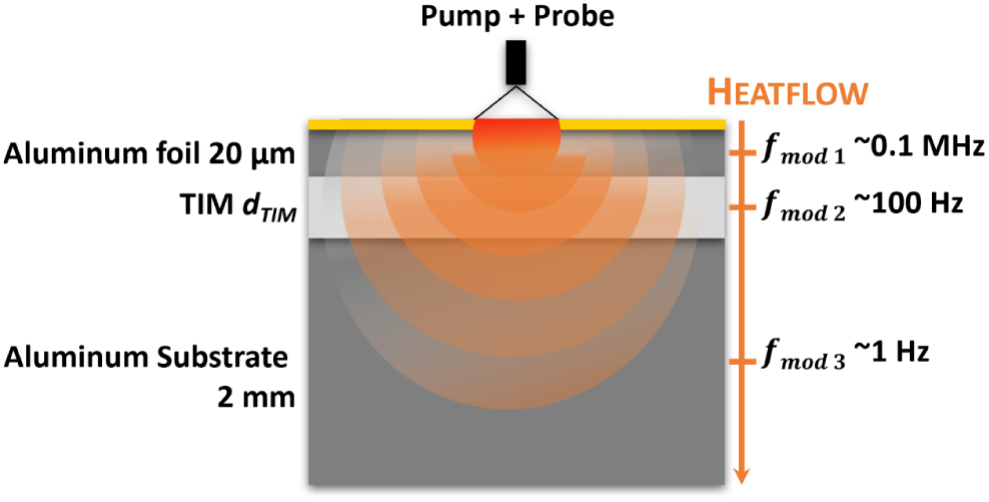
Schematic of measured samples, illustrating how the thermal penetration depth changes versus the modulation frequency (*f*_mod_). *d*_TIM_ represents the thickness of the specific TIM measured.

Two TIMs from different suppliers were measured for comparison: sample 1 is a 140 μm-thick Loctite Ablestik 5025E, a commercially available TIM film, manufactured using a silver filler, with a datasheet thermal conductivity of 6.5 W/m·K, measured using photoflash; sample 2 is a 125 μm-thick TIM film based also on silver filler, consisting of nanoparticles dispersed within an epoxy polymer matrix produced by Adamant Composites Ltd., Greece.^20^Figure 1 shows the sample structure with different TIM thicknesses (*d*_TIM_) sandwiched between a 20 μm-thick metal foil and a 2 mm-thick aluminum substrate. This metal/TIM/metal layer structure is common in many electronic device packaging applications.^26^Figure 1 also illustrates the measurement principle schematically, showing how the modulation frequency (*f*_mod_) affects the thermal penetration depth depending on the thermal properties of each layer. The thermal properties of each layer shown in Figure 1 are given in Table 1.^27^

**Table 1:** Material Properties, Including Thermal Conductivity (κ), Volumetric Heat Capacity (Cv), Thickness (*d*) for the TIM Film, and Aluminum Foil/Substrate, along with Each Parameter Uncertainty

material	κ (W/m·K)	Cv (MJ/m^3^·K)	*d* (μm)
gold transducer^a^	318 ± 3	2.49 ± 0.025	0.15 ± 0.0015
aluminum foil^a^	237 ± 2	2.43 ± 0.024	20 ± 0.4
aluminum substrate^a^	237 ± 2	2.43 ± 0.024	2000 ± 20
TIM, sample 1	4.4 ± 0.40 (fitted)	2.3 ± 0.08 (fitted)	140 ± 3
TIM, sample 2	1.2 ± 0.21 (fitted)	1.8 ± 0.21 (fitted)	125 ± 3

a The gold and aluminum properties were taken from ref.^27^.

## Heat Diffusion Model

Here, we give a brief description of the well-established heat diffusion model used to analyze the FDTR data, which is explained in detail in previous works.^17,23,24^ The thermal response in the frequency domain is given by
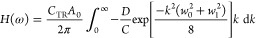
1where *C*_TR_ is the thermoreflectance coefficient of the top surface, *w*_0_ and *w*_1_ are the 1/*e*^2^ spot radius of the Gaussian pump and probe beam, respectively. *A*_0_ is the absorbed pump laser power, and *k* is the Hankel transform variable. For a multilayer structure of *n* layers, *D* and *C* are matrix elements determined by multiplying the matrices *M*_*n*_, representing layer *n* in the structure:
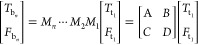
2where  and  are the temperature and heat flux on the bottom surface of the bottom layer and  and  are the temperature and heat flux on the top surface of the top layer, respectively. Each layer matrix (*M*_*n*_) contains the thickness, volumetric heat capacity, cross-plane thermal conductivity, and in-plane thermal conductivity of layer *n*.

Any unknown thermal properties, for example  and  in this case, are considered as free parameters in the heat diffusion model and are determined by using the nonlinear least-squares trust-region-reflective fitting function in MATLAB. The uncertainties of the fitted values are defined as one standard deviation, including contributions from experimental noise and the uncertainties in the controlled parameters:^28^
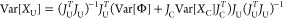
3where Var[*X*_U_], Var[*X*_C_], and Var[ϕ] are the covariance matrices of the unknow parameter vector *X*_U_, the controlled parameter vector *X*_C_, and the phase noise ϕ, respectively. *J*_U_ and *J*_C_ are the Jacobian matrices of the unknown parameter and the controlled parameter, respectively. The standard deviations of the fitted parameters are obtained by taking the square root of the diagonal elements of Var[*X*_U_].

Furthermore, it is important to note that the correlation coefficient between  and  plays a significant role in determining their respective calculated uncertainties, which is calculated using the variance  and  of each parameter:^28^
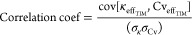
4where  is the covariance of  and .

## Measurement and Uncertainty Analysis

**Figure 2:**
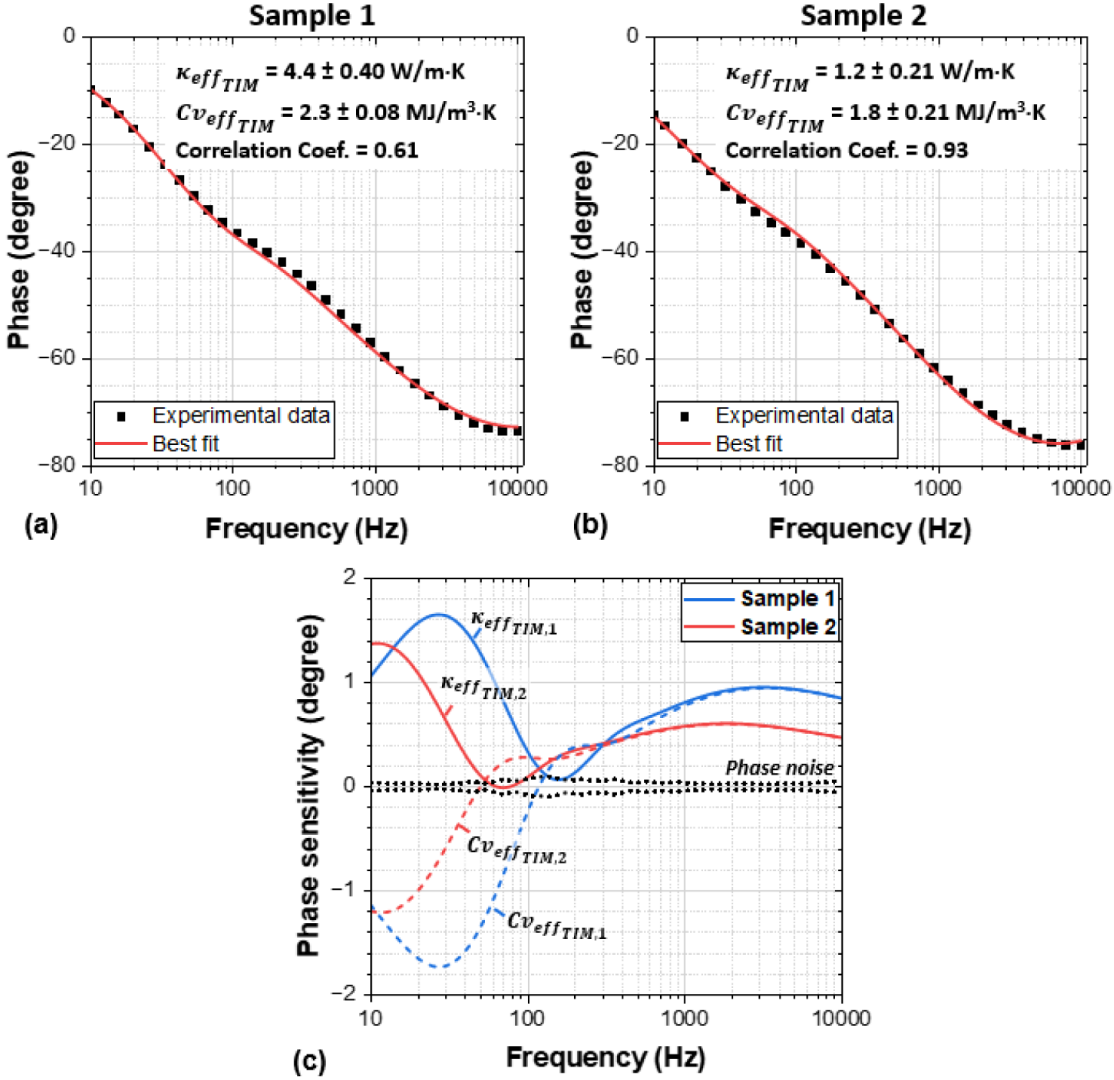
Measured FDTR phase data of sample 1 (a) and sample 2 (b), along with the best fit using the least-squares algorithm. (c) Phase sensitivities to the TIM effective thermal conductivity () and effective volumetric heat capacity () for samples 1 and 2, along with the measurements ± phase noise standard deviation.

Figure 2a,b shows modulation frequency versus phase angle plots measured for the two samples; model best-fits determined using the heat diffusion model are also plotted. The material properties obtained and their associated uncertainties are presented in Table 1 for each sample. Note that the fitted TIM thermal conductivities are effective values, including possible thermal boundary resistances (TBRs) at the aluminum foil/TIM and TIM/aluminum substrate interfaces.

As depicted in Figure 2 and outlined in Table 1, the  value determined for the commercial sample 1 is 4.4 ± 0.4 W/m·K, which is lower than the datasheet value of 6.5 W/m·K. A possible explanation is that a free-standing TIM sample was measured using the photoflash method for the datasheet thermal conductivity, which does not consider the aforementioned interface thermal resistance, whereas the sandwiched TIM (Figure 1) is more representative of a real application. This highlights possible discrepancies between standard tests used by TIM manufacturers and the TIM real-life application performances.^4−7,10−12^ The fitted  value is consistent with a silver-filled TIM film, for example, MG Chemicals silver conductive epoxy adhesive has a  of 2.2 MJ/m^3^·K. Comparing both fitted parameters of the commercial sample with the datasheet values further increases confidence of the efficacy of the low-frequency range FDTR to simultaneously fit both the  and  from a single measurement, with a reasonably low uncertainty.

Sample 2 has a lower measured  = 1.2 W/m·K than sample 1, albeit with higher measurement uncertainty, which is attributed to the higher correlation coefficient between κ_TIM_ and  for sample 2 (0.93), compared to 0.61 for sample 1. When the correlation coefficient falls within the range of 0.9–1, the correlation between  and  is considered very high,^29^ leading to increased inaccuracy in the fitted parameters.

A sensitivity analysis is a key step in determining and verifying which thermal properties are measurable and within what frequency range. The measurement sensitivity is defined as the phase difference caused by changing the parameter of interest by ±10%.^19,30^Figure 2c presents the phase sensitivity using the best fit  and  values for samples 1 and 2, illustrating that both parameters have a high sensitivity, i.e., peak values >1.2 degree, much higher than the instrument phase noise standard deviation. Since both  and  have their peak phase sensitivity at a similar frequency range, the correlation coefficient between the fitting parameters is the only way to ensure the accuracy of the fit when fitted together. This is later confirmed by Monte Carlo analysis. Note that when the TIM effective thermal conductivity decreases, the peak sensitivity to  and  decreases and shifts to a lower frequency, e.g., approaching 10 Hz for sample 2.

**Figure 3:**
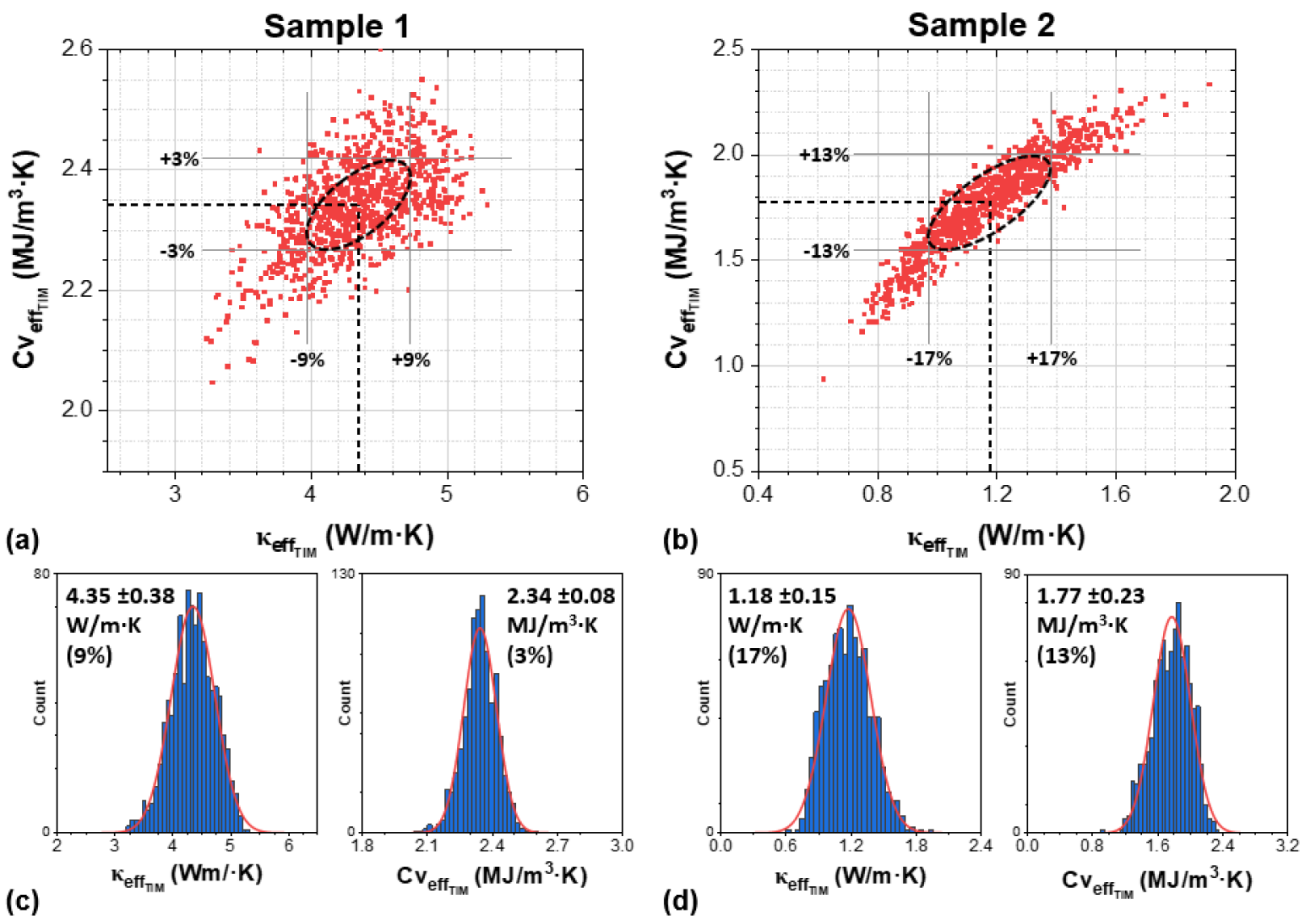
Uncertainty analysis of TIM  and . Distribution of 1000  and  fits for sample 1 (a) and sample 2 (b) using the least-squares fitting algorithm and Monte Carlo method. Each red dot represents a fitted value for both  and . Resulting histograms and fitted normal distributions for  and  for sample 1 (c) and sample 2 (d).

Monte Carlo analysis was used to rigorously check the uncertainties and correlation coefficients of the fitted  and  obtained from the standard least-squares fitting approach: the same fitting function is used, but the initial values of each controlled parameter and initial guess values of the fitted parameters are randomized. These parameters are assumed to have a normal distribution around their mean value, with uncertainties equivalent to one standard deviation,^28,31^ which are given in Table 1. Initial guesses for the fitted parameters were assumed to have uniform distribution, spanning from 0.1 to 10 times their best initial guess values, to ensure that the best-fit values correspond to a unique global minimum.^28^ The fitting is repeated 1000 times to obtain the distribution of the  and  fits. The advantage of the Monte Carlo method is that it produces robust error estimations and enables the correlation between fitting parameters to be obtained exactly. Figure 3a,b shows the distribution of the 1000 fitted  and  values for samples 1 and 2, respectively. Figure 3c,d illustrates the resulting histograms of the fitted parameters along with their normal distributions used to determine the mean and standard deviations. The value obtained from the Monte Carlo analysis agrees very well with the simpler least-squares method results shown in Figure 2a,b.

The ellipses plotted in Figure 3a,b represent ±one standard deviation contours,^31^ and their shapes indicate the correlation between  and . A larger eccentricity of the ellipse indicates a strong correlation between  and .^31^ Hence the different ellipse eccentricities for samples 1 and 2 correspondto correlation coefficients of 0.57 and 0.93, respectively. It is worth noting that the correlation coefficients calculated using the analytical method^28^ and presented in Figure 2a,b closely match the correlation calculated using Monte Carlo analysis.

**Figure 4:**
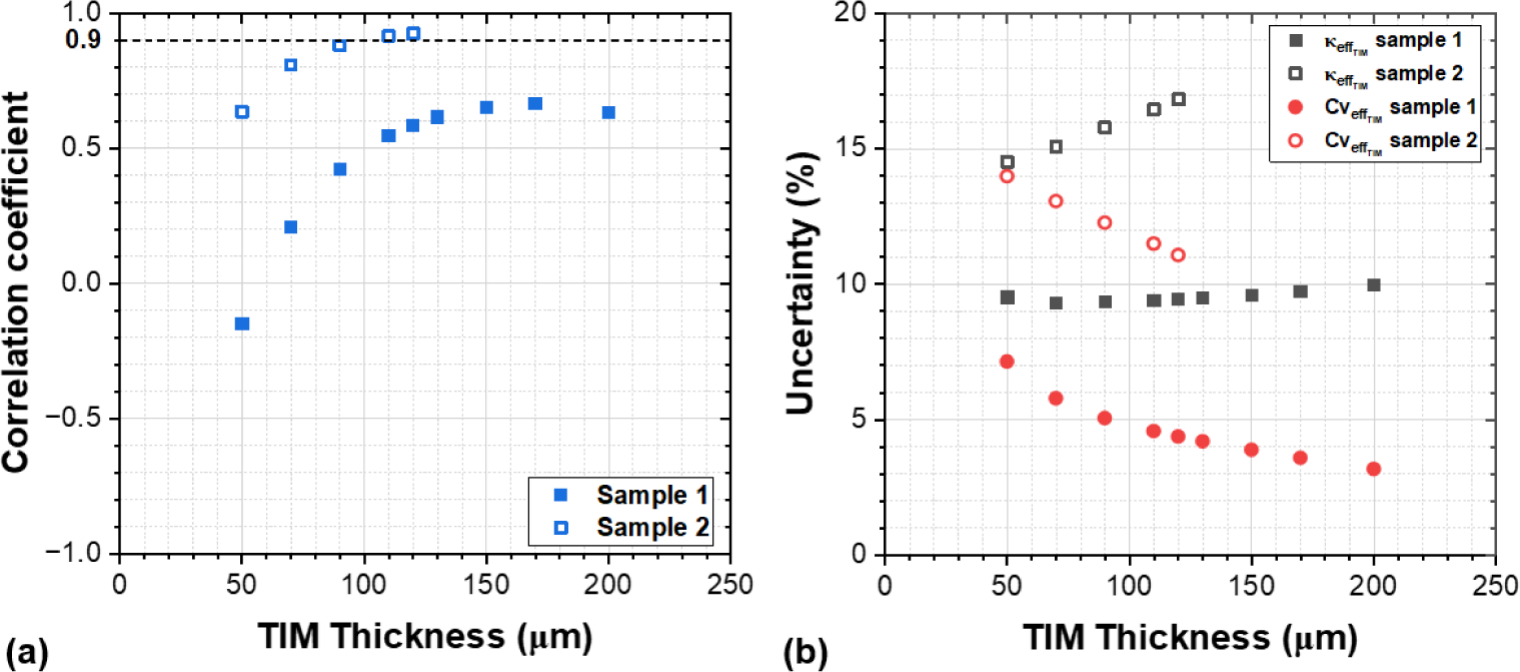
(a) Modeled correlation coefficient between  and  for sample 1 and sample 2 as a function of TIM thickness. (b)  (in W/m·K) and  (in MJ/m^3^·K) uncertainties (%) for sample 1 and sample 2 as a function of TIM thickness.

Minimizing the correlation coefficient of a particular parameter is important because it affects the uncertainty associated with both fitted parameters. To investigate this, we studied the correlation coefficients and the uncertainties of samples 1 and 2 while the TIM thickness was varied. The thermal properties shown in Table 1, including their fitted  and  values, were used to generate modeled frequency versus phase curves including synthesized measurement phase noise, which were fitted using the least-squares method described previously. The uncertainty and correlation coefficient for each case, presented in Figure 4, were calculated using eqs 3 and 4, respectively.

The thickness range upper limit studied for samples 1 and 2 corresponds to the peak sensitivity frequency of both  and  being higher than 10 Hz, i.e., the lower frequency limit of the FDTR setup.

Figure 4a shows that increasing the TIM thickness, while the TIM thermal conductivity is fixed, increases the correlation coefficient between the fitted thermal conductivity and the volumetric heat capacity. For TIMs with relatively higher thermal conductivity, such as sample 1 (4.4 W/m·K), a TIM thickness of up to 200 μm (lower measurement frequency limit of 10 Hz) can yield correlation coefficients below 0.7. However, for lower effective thermal conductivity (1.2 W/m·K) sample 2 TIM, correlation coefficients below 0.9 (cutoff for reliably fitting the effective thermal conductivity and volumetric heat capacity simultaneously) can be only achieved for thicknesses below 100 μm. This study along with the corresponding fitting uncertainties shown in Figure 4b demonstrates that, in case of sample 2, the combination of lower effective thermal conductivity and thicker TIM layer contributes to uncertainties in both the fitted  and  values being greater than 10%, higher than that of sample 1. However, we observe that the uncertainty in  decreases with TIM thickness, suggesting that reducing the measurement frequency limit <10 Hz could also be a strategy for decreasing the uncertainty for this parameter. These results highlight the impact of TIM properties on measurement uncertainties and underscore the importance of carefully considering the thickness of the TIM layer in the measurement test structure.

## Conclusions

A low-frequency range FDTR technique has proven to be highly effective in measuring the thermal properties of an in situ TIM layer. Unlike other standard methods, FDTR offers a unique capability of simultaneously determining the effective thermal conductivity and effective volumetric heat capacity of the TIM layer. This is achieved through the application of low modulation frequencies ranging from 10 Hz to 10 kHz and the choice of the test structure, where the TIM film is sandwiched between a metal foil and a metal substrate. By employing the least-squares algorithm fitting and the uncertainty analysis, validated by the Monte Carlo method, the thermal properties of the TIM can be accurately determined with measured effective volumetric heat uncertainty of less than 3–15%, depending on the TIM thickness and effective thermal conductivity. Additionally, the correlation between the fitting parameters has been investigated to provide a more accurate uncertainty determination. This capability was demonstrated through the measurement of the effective thermal conductivity and the effective volumetric heat capacity of a commercially available TIM film within a structure closely resembling a real-life application.
